# GCN2: roles in tumour development and progression

**DOI:** 10.1042/BST20211252

**Published:** 2022-03-21

**Authors:** Lyssa T. Gold, Glenn R. Masson

**Affiliations:** Division of Cellular Medicine, School of Medicine, University of Dundee, Dundee, Scotland, U.K.

**Keywords:** cancer signalling, GCN2, integrate stress response, starvation signaling

## Abstract

GCN2 (general control nonderepessible 2) is an eIF2α kinase responsible for entirely rewiring the metabolism of cells when they are put under amino acid starvation stress. Recently, there has been renewed interest in GCN2 as a potential oncotarget, with several studies reporting the development of small molecule inhibitors. The foundation of this work is built upon biochemical and cellular data which suggest GCN2 may be aberrantly overexpressed and is responsible for keeping cells on ‘life-support’ while tumours undergo significant nutritional stress during tumorigenesis, allowing cancer stem cells to develop chemotherapeutic resistance. However, most studies which have investigated the role of GCN2 in cancer have been conducted in various cancer model systems, often under a specific set of stresses, mutational backgrounds and drug cocktails. This review aims to comprehensively summarise the biochemical, molecular and cellular literature associated with GCN2 and its role in various cancers and determine whether a consensus can be developed to discern under which circumstances we may wish to target GCN2.

## Introduction

To facilitate their increased rate of proliferation, tumour cells have an elevated level of anabolic metabolism to provide sufficient material for the doubling of biomass required for cell division. Activation of oncogenic pathways may provide the stimuli which directs the cell towards growth, but in the absence of the nutrients necessary to facilitate that growth, a cancerous cell — like any cell — fails to thrive. The elevated uptake of nutrients required by cancer cells is made readily apparent through the use of radiolabelled glucose and amino acids in positron emission tomography — tumours are easily visualised by their accumulation of these nutrients. A surprising observation is that tumour cells are dependent on an exogenous supply of both essential and several nonessential amino acids [1] (reviewed [1]). Two central signalling nodes that control cellular response to amino acid availability are the protein kinases mTOR and GCN2. In this review, we will discuss the current evidence for targeting GCN2 as a cancer therapeutic.

## The ISR and GCN2

**Figure 1.**
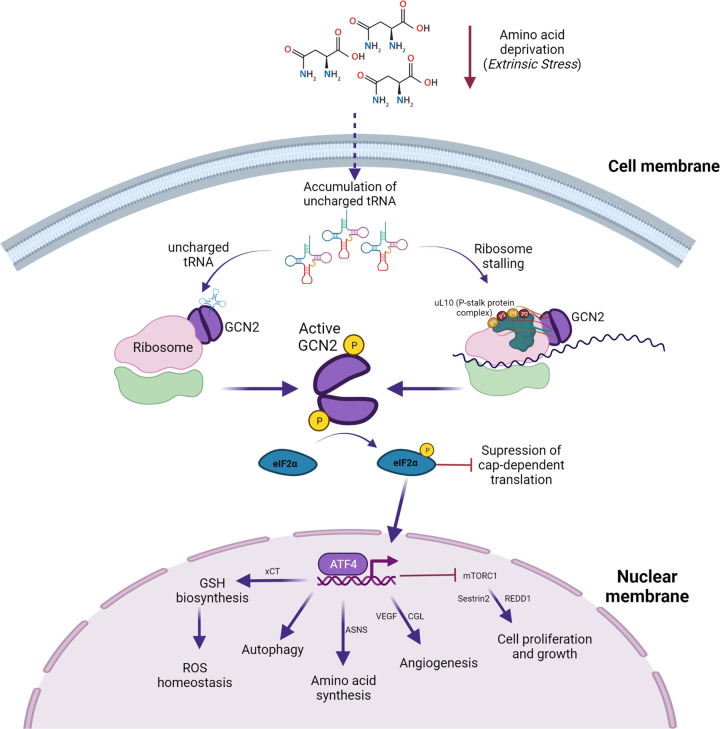
The GCN2 mediated arm of the Integrated Stress Response (ISR). On a reduction in amino acid availability, there is an associated increase in deacylated (uncharged) tRNA. GCN2 is activated through association with the ribosome, resulting in phosphorylation of eIF2α. This leads to a global reduction in cap-dependent translation, and the activation of ATF4.

The Integrated Stress Response (ISR) is a eukaryotic intracellular signalling pathway which responds to both extrinsic (i.e. amino acid or oxygen deprivation) and intrinsic (i.e. protein misfolding and proteotoxicity) stresses [2] that destabilise cellular homeostasis (see Figure 1). Stress is ameliorated by affecting changes in both global protein synthesis and the expression of certain key genes to either restore homeostasis or induce apoptosis [3]. The phosphorylation state of serine 51 on the eukaryotic initiation factor subunit eIF2α is the nexus of the response. Unphosphorylated eIF2α is necessary for translation preinitiation complex (PIC) formation and initiator Met-tRNAi ribosome interaction [4]. When phosphorylated, PIC formation is interrupted, supressing global cap-dependent protein synthesis. However, certain mRNAs possess 5′ upstream open reading frames (uORFs) that facilitate the translation of their coding region on disruption of eIF2 levels, most notably activating transcription factor 4 (ATF4) [3,5].

The four ISR serine-threonine kinases that phosphorylate eIF2α each sense a different stress: PKR-like ER Kinase (PERK) sense endoplasmic reticulum (ER) stress related to protein misfolding and is located within the ER lumen; double-stranded RNA-dependent protein kinase (PKR) responds to viral infection; heme-regulated eIF2α kinase (HRI) which senses heme deficiency; and general control non-derepressible 2 (GCN2) senses amino acid deficiency [3]. Depending on the stress type, length, or intensity, the response affected may be directly pro-survival, activating genes that oppose the infringing stress and promote a return to homeostasis, that promote cell proliferation [6] or autophagy [7], or may instead induce apoptosis if survival is not possible. Given the multiple genes under eIF2α-ATF4 control, multiple processes are likely to be influenced simultaneously in response to a single stressor to best restore cellular health.

The intrinsic and extrinsic stresses that activate the ISR in non-diseased cells undergoing stress, including metabolite deficiencies and proteotoxicity due to misfolding and aggregation, are particularly prevalent in cancerous cells which occupy the tumour microenvironment (TME) [8]. Rapid proliferation results in tumours that rapidly outgrow their blood supply, limiting access to oxygen and metabolites [9], introducing a stress state that must be overcome for cancerous cells to avoid cell death and outcompete their healthy counterparts, achieving successful, prolonged, tumorigenesis.

The ISR is one pro-survival pathway that tumours can utilise to overcome these barriers to survival and proliferate effectively, and the activity of the ISR has been demonstrated to be crucial to both the propagation and development of drug resistance in multiple solid-tumour cancers, including cisplatin resistance in gastric cancer [10], and gemcitabine resistance in pancreatic cancer [11]. The importance of the ISR and eIF2α phosphorylation in promoting cancer plasticity and metastasis can be observed using the ISR inhibitor ISRIB (Integrated Stress Response InhiBitor), a small molecule which nullifies eIF2α phosphorylation by promoting eIF2B GEF activity [12]. Breast cancers treated with paclitaxel (a first-line breast cancer chemotherapeutic), will rapidly develop resistance [13] — this can be abrogated through addition of ISRIB which re-sensitised cells to paclitaxel by preventing ISR activation. This forms the basis for investigating GCN2's role in tumour development.

## GCN2 activation in cancer

GCN2 is the only ISR kinase conserved across all eukaryotes, including yeast (Gcn2), where it is activated by a wider range of stressors [14]. Both GCN2 (and its yeast homologue Gcn2) can be activated by deacylated tRNA, which accumulate intracellularly due to amino acid scarcity. Deacylated tRNA may act as an internal indicator of amino acid deficiency and interact with the C-terminus of GCN2, specifically the HisRS-like domain which is also involved in autoinhibition of the kinase domain [15]. GCN2 may also be activated by ribosomal translational stalling [16] and the ribosomal P-stalk [17,18] but it is unclear whether these mechanisms work in unison with deacylated tRNA or whether there is a divergence in activation mechanisms between yeast and higher eukaryotic organisms.

GCN2 autophosphorylates at least two residues (T899 & T904) when activated [19] and this can act as a useful biomarker in cancer samples to determine GCN2 activation status, alongside eIF2α phosphorylation. Ye et al. [7] observed increases in both the total amount of GCN2 and phosphorylated GCN2 in colon, lung, breast and liver cancer tissue samples when compared with healthy tissue. Wang et al. [20] made similar observations in human oral squamous cell carcinoma, and, most recently, Furnish et al. [21] observed similar results in a prostate cancer study. In addition to this, a systematic quantified study determined that increased GCN2 expression levels in human papillary renal cell carcinoma were a powerful indicator of poor patient outcomes [22].

As part of a larger multiomics study Saavedra-Garcia et al. [23] investigated the available cancer bioinformatics repositories, such as the Cancer Dependency Map (DepMap) and The Cancer Genome Atlas (TCGA) to determine which cancers may be most vulnerable to GCN2 targeting [24]. They determined that ∼13% of cancer cell lines appear to be dependent on GCN2 (in comparison, they found that, 0.1%, 0% and 0.7% of the cancer cell lines screened were dependent on the other eIF2α kinases, HRI, PKR and PERK, respectively). Focusing on multiple myeloma (MM) and glioblastomas, they found that patients with tumours predicted to be GCN2-dependent (this prediction was done by looking at the transcriptional profile of patient samples available in the TCGA), had more aggressive cancers, which required earlier and sustained treatment both pharmacologically and using radiotherapy. One key commonality observed in predicted GCN2-dependent cancers was enrichment in transforming growth factor β (TGF-β) signalling genes, which may point to a functional biomarker for targeting GCN2 dependent tumours.

What is unclear is the underlying mechanism which facilitates sustained GCN2 activation in cancerous cells. GCN2 is a protein with a short half-life, and GCN2 down-regulation is most likely mediated through ubiquitination and proteasomal degradation [25], facilitated through the formation of a NEDD4L (neural precursor cell-expressed, developmentally down-regulated 4-like)-β-arrestin1/2-GCN2 ternary complex. Autophosphorylated, activated GCN2 appears to be protected from this proteasomal degradation pathway — and it is postulated that the gradual accumulation of phosphorylated GCN2 over time may drive cells to eventual CHOP mediated apoptosis. It has been suggested that it is the breakdown of this turnover pathway, rather than increased levels of GCN2 translation, is the cause for increased GCN2 levels [25]. This would suggest that the phosphatases responsible for dephosphorylating GCN2 would play a crucial role in the negative feedback mechanism of the pathway. A possible candidate is TAP42, a regulator of type 2A-related protein phosphatases [26] which once activated by TOR signalling, can dephosphorylate certain residues on Gcn2 — although there is no ready candidate for this role in mammalian systems.

In addition to this sustained up-regulation, there is function redundancy between GCN2 and PERK also. Ye et al. [7] demonstrate that both kinases phosphorylate eIF2α and contribute to ATF4 up-regulation in response to low glucose in MEFs: for GCN2 this is likely linked to glutaminolysis [27], while for PERK glucose starvation is likely to impair successful protein synthesis. This is compounded by the observation that in soft tissue sarcoma models, GCN2 KO phenotypes can be largely compensated by PERK maintaining ATF4 signalling [28] — in order to completely ablate eIF2α phosphorylation upon ISR activation it is necessary to KO both GCN2 and PERK [29]. This evidence supports GCN2's crucial role in tumour survival but also highlights a need for a further understanding of these entangled pathways if this is to be effectively utilised.

## Amino acid starvation treatment regimes

The key characteristic of GCN2 within the ISR is its role as a sensor of amino acid depletion: to respond to this effectively, amino acid synthesis and transporter genes are under control of the GCN2–ATF4 pathway. There are currently a number of amino acid depletion strategies undergoing clinical evaluation to treat various cancers, but the role of GCN2 in eliciting a therapeutic response is not always clear.

The GCN2–ATF4 pathway is crucial to the longevity of many solid-state tumours when challenged by absence of non-essential amino acids (NEAA) and their ability to oppose this stress. One well-investigated example of GCN2 inhibition coupled to an amino acid starvation treatment is that of asparaginase synthetase (ASNS), which catalyses glutamine-dependent conversion of aspartate to asparagine [30]. *In vivo*, silencing either ATF4 or GCN2 in fibrosarcoma tumours significantly reduces or entirely supresses tumour growth in mouse xenograft models [7], indicating the importance of this pathway for successful, prolonged, tumour growth even when not challenged by amino acid stress. ASNS mRNA levels, alongside amino acid transporters SLC1A4 and SLC7A5, are elevated in response to glucose deprivation, being under GCN2–ATF4 control [7]. Targeting GCN2 as a key factor in ASNS expression has already been successfully demonstrated against acute lymphoblastic leukaemia (ALL) cells, an aggressive form of blood cancer that is frequently treated with asparaginase (ASNase). Combining ASNase treatment with a GCN2 inhibitor significantly reduces proliferation in CCRF-CEM cells, with apoptosis being induced via MAPK activation, resulting in increased PARP cleavage and caspase 3/7 activity [31], indicating a sensitising relationship that could be used to augment current therapy.

Tumours utilise alternate bioenergetic pathways to supplement the energetic and catabolic cost of rapid, uncontrolled, proliferation as a response to glucose deprivation. This includes glutaminolysis, during which glutamine (Gln) is utilised by tumours in mitochondrial ATP production and as a source of catabolic carbon and nitrogen for alternate amino acid, nucleotide, and lipid synthesis [27], thus also supporting the synthesis of larger biomolecules also required for proliferation. As a precursor to other amino acids via glutaminolysis, Gln deprivation impairs this mode of amino acid replenishment in tumours, resulting in starvation, accumulation of deacylated-tRNA, and GCN2–ATF4 pathway activation. Glutamine starvation in MYC-mediated neuroblastoma results in GCN2–ATF4 activation, apoptosis, and tumorigenesis suppression [32], demonstrating the reliance of tumours on these alternate metabolic pathways, and their potential for exploitation as a therapeutic target, including via GCN2.

## ROS and protein homeostasis

In addition to ensuring metabolite supply to stressed cells, the GCN2–ATF4 pathway also removes toxic cellular products. This includes regulating oxidative stress by controlling ROS homeostasis in response to amino acid depletion [33,34]. Intracellular ROS levels increase due to mitochondrial disfunction caused by cysteine deprivation, which elevates phosphorylated GCN2 levels. This can result either in CHAC1-mediated apoptosis by glutathione (GSH) degradation[35], or GCN2–ATF4 mediated expression of xCT, the light subunit of the x_c_-cysteine/glutamate transporter, which helps maintain intracellular GSH levels [36], and protects against ROS damage caused by amino acid imbalance. The GCN2–ATF4–xCT pathway negates secondary negative effects of amino acid deprivation, however, this can also be co-opted by tumours to gain drug resistance.

The GCN2–ATF4–xCT pathway imparts cisplatin resistance in gastric cancer, and xCT is a negative prognostic indicator due to its elevation of GSH levels [10,36]. Cisplatin operates by inducing lipid peroxidation, elevating intracellular ROS levels and inducing apoptosis [10]. Elevated ROS results in GSH biosynthesis via the GCN2–xCT pathway, and the resultant cytoprotective antioxidant activity allows gastric cancer cells to resist cisplatin treatment. Inhibition of GCN2 would augment treatment in gastric cancer patients showing elevated xCT levels.

GCN2's role in ROS homeostasis may facilitate its ability to accelerate chemotherapeutic resistance development. This was made apparent through a recent and extensive transcriptome, proteome and metabolome study [23] which treated MM cells with proteasome inhibitors and then followed the changes in metabolism which facilitated recovery. It was found that inhibition of GCN2 with the inhibitor GCN2iB [31] prevented a subset of MM cells from recovering. Post-stress, GCN2 inhibition was found not only to alter amino acid levels, as expected from its canonical role, but also GSH, *N*-acetylcysteine and cysteine which indicate that GCN2 was also facilitating cellular recovery from chemotherapeutically initiated ROS stress.

Furthermore, GCN2 activity has been implicated in mitophagy [37] — the process that clears damaged mitochondria from the cell and may have a role in tumour progression [38] and the development of chemotherapeutic resistance [39]. Ghosh et al. found that even minor decreases in Mic60 (also known as Mitofilin) expression resulted in a drastic reduction in mitochondrial fitness and resulted in a rise in oxidative and starvation stress which fuelled metastasis and cell death evasion. It was shown through transcriptomics that the ISR was highly up-regulated on Mic60 depletion, with high levels of ATF4 and DDIT3 correlating with both lower Mic60 and worse patient prognosis in glioblastomas and kidney cancer. Exposing Mic60 depleted cell cultures to GCN2 inhibitors proved an effective means of selectively killing metastatic cells.

## Proteotoxicity

Tumorigenesis relies on increased protein synthesis, which can result in metabolite-related stress and subsequent protein misfolding and proteotoxicity, triggering apoptosis. Many colorectal cancers (CRC) develop loss-of-function mutations in the tumour suppressor APC (adenomatous polyposis coli), resulting in up-regulated MYC mRNA levels and proto-oncogenic activity [40], which has been shown to contribute to tumour initiation [41]. MYC up-regulates global protein synthesis, elevating amino acid and energy consumption, which in the tumour microenvironment stress state induces proteotoxic, MYC-driven apoptosis. In this context, GCN2 promotes survival by responding to the amino acid drain via a GCN2–eIF2α negative feedback loop that opposes the MYC-mediated increase in transcription via eIF2α activation, preserving metabolites, negating proteotoxic accumulation, and halting apoptosis [40]. The frequency of the APC mutations among CRC tumours and their reliance upon GCN2 for survival identifies a biomarker that can be identified for refined treatment, and a target (GCN2) for inhibition to improve therapeutic outcome. APC-deficient cells treated with the GCN2 inhibitor A-92 induces apoptosis dose-dependently, as well as decreasing eIF2α phosphorylation and elevated MYC mRNA levels [40], which as described above, would induce the observed cell death.

## Angiogenesis

Rapid tumour growth can be quickly limited by blood supply and nutrient availability (amino acids, glucose, and oxygen) [20], introducing a stress state that must either be overcome or lead to apoptosis. Tumours stimulate angiogenesis to improve nutrient availability and match blood supply to tumour growth by inducing vascular endothelial growth factor (VEGF). VEGF expression is under GCN2 control in response to amino acid deficiency (AAD) [20,42,43]. In mouse-xenografted UM-SCC-22B squamous carcinoma tumours, GCN2 knockdown supresses VEGF levels and results in a reduction in both blood vessel density and tumour volume [20], indicating that *in vivo*, GCN2's response to AAD is key to angiogenesis initiation via VEGF, a response that would usually promote vascular health in a non-cancerous state, but here promotes tumorigenesis. There is a clear relationship between GCN2 sensing and VEGF activity that could be targeted, potentially in multiple tumour cell lines.

## Cell proliferation and mTORC1

As well as responding to cellular amino acid deficiency by promoting angiogenesis, GCN2 is also able to promote cell proliferation by exerting influence over mammalian target of rapamycin complex 1, or mTORC1. Under non-stress conditions mTORC1 promotes survival and proliferation in part by up-regulating translation of mRNA's corresponding to translationally relevant proteins [6]. In healthy cells undergoing amino acid stress, these genes are down-regulated to ensure survival until homeostasis is re-established, however, during AAD, mTORC1 is under the control of GCN2, suggesting an interplay between these two amino acid sensing pathways. GCN2^−/−^ mice treated with asparaginase show a rapid increase in hepatic mTORC1 activity and subsequent mRNA translation initiation [6] and similarly in pancreatic mTORC1 under the same conditions [44], but is halted by eIF2α phosphorylation, indicating that the GCN2–eIF2α pathway exerts control over mTORC1 activity under amino acid stress, and that cell survival is promoted by eIF2α-mediated suppression of protein synthesis triggered by mTORC1.

## Potential roles of GCN1 and GCN20

Given that GCN2 activation requires an association with a heterodimer consisting of GCN1 and GCN20 (ABCF3), it could be hypothesised that changes in these proteins would likely impact on the ability of GCN2 to act as either tumour suppressor or oncogene. Recent work determining the structure of Gcn1 bound to a collided disome by Pochopien et al. [45] has provided some insight into the nature of its role in activating GCN2 (assuming that the yeast system of activation is maintained in humans). Gcn1 acts as both bridge and scaffold to straddle the collided ribosomes, interacting extensively with both the colliding and leading ribosomal P-stalks as well as their peptidyl-tRNA and deacylated tRNA. Several studies (see above) have shown how GCN1 might act as a signalling platform, allowing input from, for example, MIRO2 to facilitate GCN2 activation under a variety of stress scenarios.

The role of GCN1 in higher eukaryotic organisms may have additional complexities. GCN1 KO mice [46] exhibited distinct phenotypes when compared with GCN2 KO. While GCN2 KO mice are viable, fertile and have no discernible phenotypic abnormalities with standard growth conditions [47], GCN1 KO mice exhibit embryonic lethality due to extreme growth retardation. Furthermore, Yamazaki et al. generated mice which expressed a GCN1 mutant that lacked the RWDBD domain (Gcn1ΔRWDBD), and therefore should be unable to interact with GCN2. Gcn1ΔRWDBD mice had a distinct phenotype, with a milder growth defect and perinatal lethality most likely due to abnormalities in lung development. It may be that the role of GCN1 in facilitating ribosomal recycling may explain the more dramatic phenotype of its loss. Furthermore, a recent study implicated GCN2 activity as a sensor of ribosomal fitness [21]. This study focused on mitochondrial Rho GTPase 2 (*MIRO2*) — a prognostic marker for metastatic prostate cancer and found that MIRO2 interacted directly with GCN1 and this interaction facilitated GCN2 kinase signalling and ATF4 activation. In addition to this, MIRO2 expression levels correlated with both GCN2 and ATF4 expression levels and levels of phosphorylated GCN2 in prostate cancer xenograft models. As MIRO is a mitochondrial protein, located within the outer mitochondrial membrane, it may serve as a sensor of mitochondrial health and activate GCN2 in response to ROS.

While little is known on the role of GCN1, less is known about GCN20 (ABCF3). ABCF3 has been associated with both chemotherapeutic resistance in ovarian cancer [48] and enhances human liver cancer cell line proliferation [49].

## Conclusion

There appears to be a number of contradictions on how best to target GCN2 as a therapeutic in cancer. Firstly, while it may appear that inhibiting GCN2 would appear to prevent chemotherapeutic resistance and angiogenesis and would stunt the rapid proliferation of cancer cells, especially those tumours that are dependent on certain amino acids to proliferation. However, GCN2's role in promoting apoptosis under a number of conditions would point towards the selective activation of GCN2 being a route to therapy. Secondly, although GCN2 appears to have a driving role in a number of different types of cancer, such as ovarian cancer, leukaemia, glioblastoma and MM, but this appears to only be of consequence under certain mutational backgrounds.

Perhaps the best characterised role for GCN2 is as a facilitator of chemotherapeutic resistance. By allowing cells to rewire their metabolism after exposure to chemotherapeutic agents, it facilitates survival in certain populations of cells which then go on to metastasize. It is likely here where there will be the greatest interest in developing a GCN2 inhibitor which can be used in combination with first line therapies, to prolong their potency.

## Perspectives

- A near-universal hallmark of early tumours and cancers is a persistent state of nutrient starvation. As a critical node in managing this starvation stress, GCN2 appears to be an attractive and possibly selective therapeutic cancer in a broad spectrum on cancers.
- Currently, there are two possible routes to GCN2 mediated cancer therapy – activation induced apoptosis and inhibition of GCN2 to sensitise tumours to amino acid starvation. It is not clear in which context is the preferred option.
- Both fundamental aspects of GCN2 regulation and the role of GCN2 in specific human tissues need further exploration in order to develop GCN2 targeting therapy.

## Open Access Statement

Open access for this article was enabled by the participation of University of Dundee in an all-inclusive *Read & Publish* agreement with Portland Press and the Biochemical Society under a transformative agreement with JISC.

## Abbreviations

AAD: amino acid deficiency
ASNS: asparaginase synthetase
CRC: colorectal cancers
GSH: glutathione
HRI: heme-regulated eIF2α kinase
ISR: integrated stress response
ISRIB: integrated stress response inhibitor
MIRO2: mitochondrial Rho GTPase 2
MM: multiple myeloma
PERK: PKR-like ER Kinase
PIC: preinitiation complex
TCGA: The Cancer Genome Atlas
VEGF: vascular endothelial growth factor
